# Estimation of reproduction numbers in real time: Conceptual and statistical challenges

**DOI:** 10.1111/rssa.12955

**Published:** 2022-11-22

**Authors:** Lorenzo Pellis, Paul J. Birrell, Joshua Blake, Christopher E. Overton, Francesca Scarabel, Helena B. Stage, Ellen Brooks‐Pollock, Leon Danon, Ian Hall, Thomas A. House, Matt J. Keeling, Jonathan M. Read, JUNIPER Consortium, Daniela De Angelis

**Affiliations:** ^1^ Department of Mathematics The University of Manchester Manchester UK; ^2^ Joint UNIversities Pandemic and Epidemiological Research UK; ^3^ The Alan Turing Institute London UK; ^4^ MRC Biostatistics Unit, School of Clinical Medicine University of Cambridge Cambridge UK; ^5^ Statistics Modelling and Economics Department Public Health England London UK; ^6^ Joint Modelling Team Public Health England London UK; ^7^ Manchester University NHS Foundation Trust Manchester UK; ^8^ Department of Physics Humboldt University of Berlin Berlin Germany; ^9^ Department of Physics and Astronomy University of Potsdam Potsdam Germany; ^10^ NIHR Health Protection Research Unit (HPRU) in Behavioural Science and Evaluation, Population Health Sciences University of Bristol Bristol UK; ^11^ Department of Engineering Mathematics University of Bristol Bristol UK; ^12^ School of Health Sciences The University of Manchester Manchester UK; ^13^ Mathematics Institute and School of Life Sciences University of Warwick Coventry UK; ^14^ Centre for Health Informatics, Computing and Statistics, Lancaster Medical School Lancaster University Lancaster UK

**Keywords:** growth rate, real‐time estimation, reproduction numbers

## Abstract

The reproduction number R has been a central metric of the COVID‐19 pandemic response, published weekly by the UK government and regularly reported in the media. Here, we provide a formal definition and discuss the advantages and most common misconceptions around this quantity. We consider the intuition behind different formulations of R, the complexities in its estimation (including the unavoidable lags involved), and its value compared to other indicators (e.g. the growth rate) that can be directly observed from aggregate surveillance data and react more promptly to changes in epidemic trend. As models become more sophisticated, with age and/or spatial structure, formulating R becomes increasingly complicated and inevitably model‐dependent. We present some models currently used in the UK pandemic response as examples. Ultimately, limitations in the available data streams, data quality and time constraints force pragmatic choices to be made on a quantity that is an average across time, space, social structure and settings. Effectively communicating these challenges is important but often difficult in an emergency.

## 1 INTRODUCTION

Real‐time assessment of the current state and trend of an evolving pandemic is vital for situational awareness, evaluation of the impact of policies and guidance for the choice and timing of further interventions, all of which have been highlighted during the UK COVID‐19 pandemic. These assessments either provide snapshots of the epidemic state (e.g. infection prevalence, hospital occupancy) (Survey, 2021) or describe epidemic trends (e.g. growth rate, reproduction number) (Anderson et al., 2020). The combined analysis of the current state and onward trend is required to better inform the introduction or relaxation of public health interventions (Brooks‐Pollock et al., 2021). Despite this, politicians, media and the public predominantly have focused on a single measure, resulting in the reproduction number becoming the headline of the COVID‐19 pandemic in many countries worldwide (e.g. in the United Kingdom, it is a key value reported weekly by the Government, UK Government Guidance, 2020). Such focus risks putting too much emphasis on a single number, potentially obscuring heterogeneity, and highlights the need for transparency on the complexities involved in its real‐time estimation and interpretation as a key epidemiological indicator.

In this work, we consider three different reproduction numbers, $R_{0}$, $R_{e}(t)$ and $R_{c}(t)$, the basic, effective and control reproduction number, respectively.

The *basic reproduction number* $R_{0}$ describes the expected number of secondary infections caused by a typical infected individual in a completely susceptible host population, under standard behavioural patterns. $R_{0}$ is thus a constant which aptly describes the early stages of an epidemic caused by a novel pathogen. As the epidemic spreads fewer secondary infections may be caused, either due to behavioural changes or a reduction in the susceptible pool due to, for example, prior infection or death. These two drivers motivate the introduction of $R_{e}(t)$ and $R_{c}(t)$.

**FIGURE 1:**
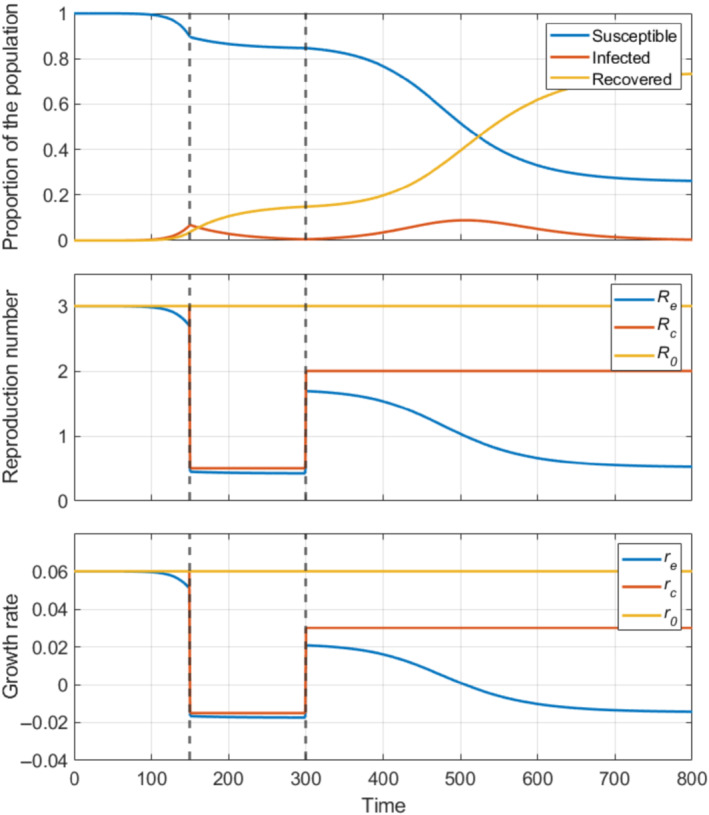
Illustrative example of dynamics, reproduction numbers and growth rates for a simple epidemic model (Section 2.1) with an average infectious period of 30 units of time. We let the transmission rate drop suddenly at time 150 and increase again, but not up to the initial value, at time 300 (dashed lines) to simulate the implementation and partial relaxation of a non‐pharmaceutical intervention limiting mixing (e.g. a lockdown). Note that all three reproduction numbers coincide when there are no interventions and the depletion of susceptibles is negligible. [Colour figure can be viewed at wileyonlinelibrary.com]

The *effective reproduction number* $R_{e}(t)$ (often denoted by $R_{t}$) describes the expected number of secondary infections under the current conditions of population mixing, transmission and immunity. Figure 1 illustrates how both drivers are captured by a smooth decline as the pool of susceptibles decreases, and in response to interventions that impact mixing.

The *control reproduction number* (or *reproduction number excluding immunity*), $R_{c}(t)$, describes the expected number of secondary infections under the current contact and transmission patterns in an otherwise fully susceptible population. Figure 1 illustrates its time‐variation resulting from an intervention aimed at reducing transmission that is implemented on day 150 and partially relaxed on day 300, without the smooth changes resulting from the depletion of susceptibles. $R_{c}(t)$ therefore does not describe ongoing transmission, except when immunity in the population is negligible. However, it allows comparison of the effects of control measures applied at different times in the epidemic by factoring out the impact of different levels of immunity at those times, and has been fundamental in the discussions guiding or estimating the impact of control policies during the highly variable intervention timeline of the COVID‐19 pandemic.

One of the most common uses of these reproduction numbers (generically denoted by R) is related to their threshold value of 1: $R_{0}>1$ means that the infection can cause a large outbreak in a fully susceptible population; $R_{e}(t)>1$ indicates that the epidemic is growing under the current conditions; and $R_{c}(t)>1$ means that the control measures alone are insufficient to contain an invasion in a susceptible population, although the epidemic may be effectively decaying due to widespread immunity.

An alternative quantity characterising the dynamics of an epidemic is the real‐time growth rate r, which, in contrast to a reproduction number, describes the exponential rate at which new infections grow (r>0) or decline (r<0). In what follows we distinguish between the basic ($r_{0}$), effective ($r_{e}(t)$), and control ($r_{c}(t)$) growth rates, associated with the three reproduction numbers above.

The simplicity of these definitions is invaluable for guiding intuition, and they ignore many real‐life complexities. For example, $R_{0}$ depends both on the pathogens and on the natural transmission patterns (biological and behavioural) in the population, so although here described as constant, it might be different in different populations, settings and environmental conditions (e.g. when seasonality affects virus survival and hosts' natural contact patterns ‐ see remarks in Section 2.1).

In this paper, we discuss the challenges, both theoretical and practical, that arise when defining reproduction numbers in models of increasing complexity and when estimating them in practice. We additionally provide examples of such challenges for models used by research groups in the Scientific Pandemic Influenza Group on Modelling (SPI‐M), informing the official estimates published by the UK Government in response to the current COVID‐19 pandemic. In parallel we also consider growth rates, which historically have preceded reproduction numbers, more naturally emerge from the representation of epidemic as dynamical systems, and are easier to estimate. We conclude with a discussion of the dominant role that reproduction numbers have played, and their strength and limitations. We argue that, ultimately, no single measure in isolation is sufficient to guide policy response, so multiple metrics should be used in combination.

## 2 DEFINITION AND PROPERTIES OF REPRODUCTION NUMBERS IN SIMPLE EPIDEMIC MODELS

A common feature of many epidemics and epidemic models is the presence of an exponentially growing phase, that is, a time window during which infections are growing or declining exponentially. Such a phase occurs whenever the depletion of the susceptible population as the epidemic progresses can be ignored, that is, when the system's dynamics can be reasonably approximated with a linear process. The most commonly considered case is in the early phase of the epidemic, when the number of infected, though growing, does not change too rapidly in absolute terms. In this phase, the epidemic will grow if $r_{0}>0$, and will die out if $r_{0}<0$.

The same epidemic can be followed in a 'generational' perspective (Keeling & Rohani, 2011, chapter 2). In this case, during the same exponentially growing phase, the number of cases will grow geometrically from one generation to the next (being this a discrete‐time linear process with a generation, i.e. the average time from one infection to the infectious contacts they make, as time unit), with per‐generation multiplicative factor $R_{0}$. The threshold condition $r_{0}=0$ above is therefore equivalent to $R_{0}=1$. Note that this change of perspective, from continuous time to generations, ‘removes’ time from the epidemic process: without additional information on the duration of a generation, $R_{0}$ cannot describe the speed of the infection; conversely, the real‐time growth rate $r_{0}$ cannot provide direct information on the number of infections generated by each infected individual.

With small adjustments, the same concepts apply to their stochastic formulation: a branching process provides the linear approximation to the early phase of the epidemic, which in the case of a discrete‐generation perspective is a Galton–Watson branching process with $R_{0}$ being the mean of the offspring distribution (Diekmann et al., 2012, chapter 1).

In the following sections, we consider some well‐studied deterministic models and discuss the definitions of $R_{0}$ and $r_{0}$ in each case.

### 2.1 Single‐type, homogeneously mixing models

In the case of the well‐known deterministic Susceptible‐Infected‐Recovered (SIR) model (Keeling & Rohani, 2011, chapter 2) with transmission rate β and recovery rate γ, the linear approximation to the epidemic process is described by

(1) $$\frac{dI}{dt}=\left(\beta -\gamma \right)I,$$

where I(t) denotes the total number of infected (and infectious) individuals at time t, and the growth rate, or *Malthusian parameter*, is $r_{0}=\beta -\gamma$ (Diekmann et al., 2012, chapters 1 and 8.2). In the generational perspective, $R_{0}=\beta /\gamma$ and the time unit of the discrete process is 1/γ. Note that as β and γ are both rates, their ratio is dimensionless.

This linear approximation is typically employed in the early epidemic phase, but can also be applied at any point of the epidemic if the considered time window is sufficiently short (and giving, in the limit of an infinitesimal window, the instantaneous epidemic trend). In the latter case, depletion of susceptibles may be relevant, and one should use $r_{e}(t)=\beta S(t)/N-\gamma$ and $R_{e}(t)=R_{0}S(t)/N$ in place of $r_{0}$ and $R_{0}$, where S(t) denotes the number of susceptible individuals at time t.

In the simplest epidemic models, basic parameters (in particular β) are assumed constant. However, in practically relevant contexts, they are likely to change in time, either as a consequence of imposed controls, or due to natural variation of environmental conditions. For example, the aggressive implementation of physical distancing measures (e.g. lockdown) during the COVID‐19 pandemic inverted the initial growth into a decline. The latter was not driven by depletion of susceptibles, but rather by a change in contact patterns. In this context, the transmission rate in (1) would be described by a time‐dependent function β(t), while the control reproduction number $R_{c}(t)=\beta (t)/\gamma$, and the control growth rate $r_{c}(t)=\beta (t)-\gamma$, capture the changes in transmission.

When parameters change due to natural variation over time in environmental conditions affecting virus survival and fitness, behaviour of vectors (if present) or natural contact patterns in the population, for example, weather and school terms, $r_{0}$ and $R_{0}$ should be treated as time‐dependent quantities. However, in the particular example of periodic time variations, like simple representation of seasonality, an alternative definition of $R_{0}$ that maintains its desirable feature of being a single number can be obtained by integrating a time‐varying $R_{0}$ over the entire period (Bacaër & Guernaoui, 2006). The usefulness of such a definition, though, depends on the particular question at hand and, in particular, the time‐scale of the epidemic compared to the duration of a full period: averaging over a yearly seasonal oscillation in transmission might be informative for infectious period lasting decades (e.g. HIV), but might not be helpful when an epidemic is consumed throughout a single winter season (e.g. influenza or measles).

Variations to the simplest model involving for instance additional latent phases or multiple compartments in series or parallel are conceptually similar, though simple formulae and analytic results are quickly lost as the complexity increases.

A mathematically different generalisation, formulated with integral equations or partial differential equations, is obtained when the infectivity is described as a function β(τ), where τ denotes the time since infection. The same concepts as in the model with constant recovery rate apply, but now $R_{0}=\int_{0}^{\infty }\beta (\tau )d\tau$ and $r_{0}$ is the implicit solution of the characteristic (or Euler–Lotka) equation (Diekmann et al., 2012, chapters 2 and 9)

(2) $$\int_{0}^{\infty }\beta (\tau )e^{-r_{0}\tau }d\tau =1.$$

The *generation time distribution* (or *generation interval distribution*), that is, the distribution of time intervals between the infection of a case and the transmission events they make, is given by $\omega (\tau )=\beta (\tau )/R_{0}$. Like 1/γ in the model with constant recovery rate, ω(τ) is the key piece of temporal information that, through the Euler–Lotka equation (2), links the real‐time epidemic perspective with the a‐temporal generation perspective. The same considerations above in the case of time‐varying environmental conditions can be applied to this model, with a transmission rate β(t,τ) that is allowed to vary over both calendar time and time‐since‐infection, and hence a basic reproduction number that can vary with time.

### 2.2 Multi‐type models

When individuals are distinguished in classes or sub‐populations that are epidemiologically distinct from each other, for example different age bands or geographical locations, the model is typically referred to as a multi‐type model. Individuals of each type are assumed to be epidemiologically homogeneous, and each sub‐population is assumed to be large. If there are m types, the transmission rate β in (1) is replaced by an m×m matrix $B=\left(\beta_{ij}\right)$, where $\beta_{ij}$ describes the transmission rate from each type j to each type i (Keeling & Rohani, 2011, chapter 3) and the linear equation in (1) is replaced by a system of m linear equations for the infectious individuals of each type i, $I_{i}(t)$. The system is described by the matrix $H=B-\gamma J_{m}$ ($J_{m}$ being the m‐dimensional identity matrix). Note that, like in the single‐type case, the total number of cases will eventually grow exponentially with rate $r_{0}$. However, in this case $r_{0}$ is computed as the dominant eigenvalue (or spectral radius) of H and the exponential growth only occurs after some transient dynamics, dependent on the initial conditions, required for the proportion of cases of each type to converge to the elements of the (normalised) right eigenvector relative to $r_{0}$. In this case, the exponentially growing phase consists of the window after the initial transient dynamics but before any of the sub‐populations starts experiencing depletion of susceptibles.

From a generational perspective, the dynamics are described by the next generation matrix $K=\left(k_{ij}\right)=\left(\beta_{ij}/\gamma \right)$, where $k_{ij}$ gives the number of cases of type i infected by an infected individual of type j in a fully susceptible population (Diekmann et al., 2012, chapter 7). The same caveats about initial transient dynamics apply, and $R_{0}$ is defined as the dominant eigenvalue of K, which gives the per‐generation multiplicative factor of an epidemic when individuals are distributed in types proportionally to the right eigenvector relative to $R_{0}$. The equivalence of the threshold conditions $r_{0}=0$ and $R_{0}=1$ holds also in the more complex case when γ is type‐dependent, or when a multi‐type time‐since‐infection model is used and $B=\left(\beta_{ij}(\tau )\right)$. The existence of $r_{0}$ and $R_{0}$ (as unique and real largest eigenvalues) for biologically sensible matrices H and K (i.e., with non‐negative elements) is typically guaranteed by Perron–Frobenius theory (Diekmann et al., 2012, chapter 7) under the technical conditions of *primitivity* and *irreducibility*. Notable cases that require special attention are for instance vector‐borne diseases (matrices are not primitive), and cases where the epidemic remains ‘contained’ in some sub‐populations, so that some types are never infected (matrices are reducible). In practice, complications arise when the matrix is ‘almost’ reducible, for example in the case of an epidemic spread in different geographical locations with very weak coupling between locations. In this situation, with a finite population, depletion of susceptibles in various sub‐populations may occur before the system converges to the dominant eigenvector, thus effectively compressing the exponentially growing phase to the point it disappears, and resulting in de‐synchronised epidemics.

### 2.3 Properties of $R_{0}$

In the simple SIR model (Keeling & Rohani, 2011, chapter 2), in addition to being a threshold parameter and informing the amount of transmission to be blocked to achieve control, $R_{0}$ has two further properties: (a) the final epidemic size depends only on $R_{0}$; and (b) $R_{0}$ directly provides the critical immunity threshold $c_{T}=1-1/R_{0}$ (Keeling & Rohani, 2011, chapter 8). These two highly praised properties, however, are only true in the simplest single‐type model. Already with multi‐type models, $R_{0}$ no longer describes the final size (Diekmann et al., 2012, chapter 1), though it still informs the critical vaccination coverage if the vaccine is distributed at random (better strategies, however, are possible, which are not fully characterised by $R_{0}$ alone). As soon as the model includes local saturation of susceptibles, the second property is also generally lost, for example, in the case of households models $1-1/R_{0}$ only provides a lower bound to $c_{T}$ (Ball et al., 2016).

Further complications arise when $R_{0}$ is allowed to vary with time, for example, due to natural time variations in parameters, as the critical immunity threshold will also be time‐dependent. In the case of time variations imposed by interventions, instead, we interpret the critical immunity threshold as referred to the natural (pre‐intervention) population mixing, and therefore $R_{c}(t)$ alone cannot inform the critical vaccination threshold: for example, if $R_{0}=4$ and, at a certain time t, $R_{c}(t)=2$, the epidemic would appear to be declining if 50% of the population is immunised. However, lifting non‐vaccine‐based controls would lead to epidemic growth when the immune population is less than 75%.

## 3 ESTIMATING REPRODUCTION NUMBERS

The reproduction numbers R can be directly estimated in real time from cohorts of infected individuals if knowledge is available on chains of transmission (e.g. who infected whom). This information is hardly ever available, so the R values need to be estimated using an underlying mathematical model linking transmission to other epidemiological information. A rich literature exists exploiting time series data on reported infections, including methods: based on the early exponential growth of an outbreak (Lipsitch et al., 2003); using information on symptoms onsets of reported cases over time and on the generation time distribution (see Section 2.1) to reconstruct the unobserved chains of transmission (Wallinga & Teunis, 2004) and derive an estimate of R; generalising the Euler–Lotka equation (2) and coupling it with estimates of the generation time distribution to derive R through the estimation of the underlying number of infections (Cori et al., 2013). These all depend on: consistent (over time) and unbiassed (i.e. not subject to ascertainment biases) collection of the data they use; knowledge or ability to estimate the generation time distribution or its proxies (e.g. serial interval); and in some cases on the ability to reconstruct the unobserved underlying number of infections from the available data. These issues have all been discussed in recent literature (Britton & Scalia Tomba, 2019; Gostic et al., 2020).

Alternatively, estimates of R can be derived from mechanistic models of transmission (Anderson et al., 2020, table A1). Here the first practical challenge is again that transmission events are not observed, therefore the compartmental structure (e.g. S and I) is not informed by direct data. So, the approach is to consider the model compartments as latent and estimate the parameters governing the system dynamics by linking indirect data to these compartments. For COVID‐19, the main surveillance data sources available are symptomatic cases, hospitalisations and deaths (UK Government, 2021), supplemented by randomised swabbing (REACT 1, 2020; Survey, 2021) and serology (REACT 2, 2020). The linking between observations and the unobserved transmission process is carried out through the specification of observational models. For instance, hospitalisations result from infections developing severe symptoms and being hospitalised. Assuming some information about the probability of hospitalisation given infection and the delay between infection and hospitalisation, theoretical hospitalisations can be derived from the transmission model. The observed data are then treated as an imperfect measure of the modelled hospitalisations and the observational model is constructed to reflect this, by assuming an appropriate error model. In a likelihood‐based inference using multiple data streams, a likelihood component is specified for each stream as a function of all the parameters leading to the observed data, including the unknown parameters governing the infection process. Then maximisation of the likelihood or its use in a Bayesian framework allows estimation of parameters and any of their functions (e.g. reproduction numbers) (De Angelis & Presanis, 2019).

In what follows we report on our direct experience of estimation of R using a non‐mechanistic model of the type of Cori et al. (2013), as well as single and multi‐type deterministic mechanistic models.

### 3.1 Examples and challenges from the UK response

Over the course of the pandemic, modelling groups from a number of UK universities and public bodies have routinely supplied real‐time estimates of the current state of the pandemic to SPI‐M. Of the various modelling outputs, estimates of $R_{e}(t)$ became the headline figure used to inform policy and communicate the severity of the pandemic to the public. In this section we look at how these estimates were produced by a subset of the contributing modelling groups.

#### 3.1.1 Non‐mechanistic $R_{e}(t)$ estimation

Multiple groups (e.g. Bristol/Exeter, Lancaster) provide near real‐time $R_{e}(t)$ estimates for SPI‐M, using an implementation of the Cori method (Cori et al., 2013; Thompson et al., 2019), which only models one generation of transmission without requiring a full mechanistic formulation of the transmission process. The method estimates $R_{e}(t)$ from

$$R_{e}(t)=i(t)/\overset{t}{\underset{s=1}{\sum }}i(t-s)w_{s},$$

where i(t) is the infection incidence at time t, and $w_{s}$ is the discretised serial interval distribution, measured from the symptom onset data between pairs of epidemiologically linked cases and used as a proxy for the generation interval distribution ω(s) indirectly used in (2). The Cori method is widely used, and the challenges surrounding the resulting $R_{e}(t)$ estimates mainly relate to data quality, data processing, the appropriate spatial scale at which to aggregate case data, and interpretation. Groups differ in their choice of input data and the processing of those data. Data include NHS 111 (emergency) calls, testing data, hospital admissions and deaths, each with specific time intervals from infection, often estimated from limited observations. The Cori method requires information about the generation time distribution, and groups used estimates from different studies (see e.g. Challen et al., 2021). The generation time is likely to be population‐specific and change with public health interventions and behavioural responses (Challen et al., 2020); estimates for the United Kingdom were not available until July 2020 (PHE, personal communication). The Cori method estimates $R_{e}(t)$ over a subjectively chosen rolling time window during which the effective reproduction number is assumed to be constant (Cori et al., 2013). Window sizes differ between groups, ranging between 5 and 28 days (Challen et al., 2021). Other methodological issues associated with data processing, to account for right censoring (e.g. individuals who are infected but have yet to develop symptoms and be tested or admitted) and how best to impute infection dates (from data anchored to testing date, for example) are covered elsewhere (Gostic et al., 2020). As with other approaches, the suitability of estimating $R_{e}(t)$ using this method at large geographical scales depends on assumptions about mixing and transmission within the population. Changes in data collection, spatio‐temporal variation in testing rates or care‐seeking behaviour were generally not accounted for, due to limited data being available to estimate how these processes vary over time and space. Because of the relatively short delay between infection and emergency calls or testing data compared to other data streams, $R_{e}(t)$ estimates from the non‐mechanistic approaches and testing data often provided more up‐to‐date estimates than other approaches. However, because the method does not assume an underlying transmission process, the resulting estimates are effectively only a transformation of the (reconstructed) incidence curve, so cannot disentangle the impact of immunity from a decrease in transmission (i.e. an estimate of $R_{c}(t)$ is not possible), or forecast epidemic dynamics beyond the most recent data point.

#### 3.1.2 Manchester/Oxford/Lancaster model

This is an example of a single‐type compartmental model with multiple latent and infectious compartments, coupled with a detailed description of progression through different hospital compartments. The model is calibrated, through a Bayesian approach, using time‐series data for daily hospital admissions, daily deaths in hospital, and the total number of beds occupied and patients in critical care, as well as information on hospital length of stay for COVID‐19 patients (Vekaria et al., 2021). The out‐of‐hospital component of the model, which describes transmission in the community, is informed by estimates of incubation period, generation time and severity from the literature, with only the most severe cases seeking hospital admission. Further details are reported in Anderson et al. (2020) and the full model description can be found in Overton et al. (2021).

**FIGURE 2:**
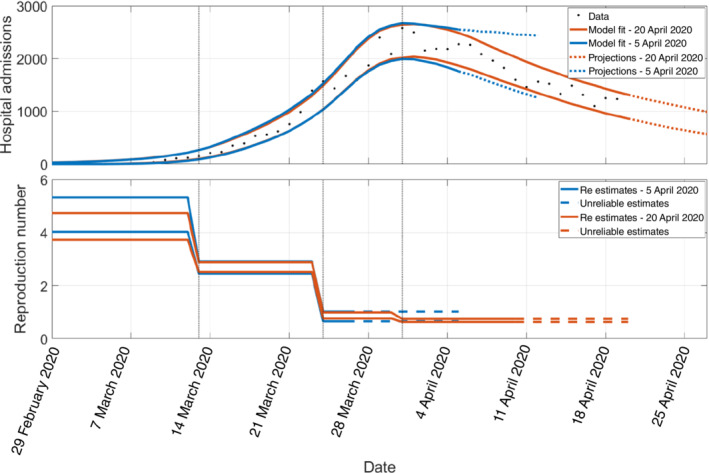
Illustration of Manchester/Oxford/Lancaster model fit to COVID‐19 hospital admissions 2 weeks (blue) and 4 weeks (red) after the first UK lockdown. Notice how: a change in transmission only becomes visible in hospitalisations more than a week later; the noise in the data makes it difficult to estimate a robust trend well after the peak has passed; uncertainty in $R_{e}(t)$ estimates depends on how many data points are available since the last allowed change in transmission (2 weeks for blue, 3 for red); and how past estimates of the effective reproduction number change retrospectively as more data become available. The paired lines indicate the 95% prediction interval in the top panel and 95% confidence interval in the bottom panel. The vertical dotted lines indicate when transmission rate changes: the first date (13/03/2020) corresponds to 9 days before a clear change in the log‐scale admissions gradients; the second (23/03/2020) to the first lockdown in the United Kingdom; and the third (31 March 2020) is a flexible change point that is added three weeks before the last data point (provided no major changes occurred in this 3‐week window), in order to capture more recent changes in transmission. [Colour figure can be viewed at wileyonlinelibrary.com]

With behavioural changes brought about by policy, a time‐varying transmission rate needs to be allowed in the model (Figure 1 and Section 2.1). This model assumes that the transmission rate β(t) is piecewise constant, with change points fixed to days of important interventions or clearly visible changes in data trends. A final change is allowed 3 weeks before the last data point, which is short enough to provide a relatively up‐to‐date estimate of the reproduction number (an average over the last 3 weeks) but long enough that noise in the data does not excessively affect the point estimate. Since this model only uses hospital data and it takes on average about 10 days from infection to hospitalisation, although $R_{e}(t)$ is averaged over the last 3 weeks in the model, the impact of any significant event likely to influence transmission within the last 10 days would not be detected (Figure 2).

The fact that the model is only fitted to hospital data means there is a lack of identifiability in the out‐of‐hospital epidemic (in the absence of reliable prior parameter estimates on the hospitalisation rate). For instance, the same hospital data would be fitted equally well by two different epidemics, one in which twice as many individuals get infected as in the other, but only half as many are hospitalised. Since $R_{e}(t)$ is strongly informed by the observed growth rate in hospital admissions (irrespective of the amount of immunity), these two epidemics will be associated with the same $R_{e}(t)$. However, the $R_{c}(t)$ values will differ because one epidemic is characterised by a higher transmission rate (but smaller susceptible population) compared to the other. Therefore, such a model will generate reliable estimates for $R_{e}(t)$, but may struggle to accurately estimate $R_{c}(t)$ in the absence of informative priors.

Finally, this model does not consider age structure in the population, which is simultaneously an advantage and a limitation. For example, without age structure, the reproduction number is easily defined and understood within this model (Section 2.1 and Figure 1). However, the reproduction number estimates may not reflect the real transmission trends in periods where the distribution of cases across age groups is not stable. In such periods, age‐structured models can provide an advantage, but might also suffer from problems related to convergence to the dominant eigenvector (Section 2.2 and 3.1.3). Finally, irrespective of whether the model has age structure or not, models purely informed by data on severe events may be unable to pick up growth among cases in children (e.g. when only schools reopen after a lockdown), if their infections are much less severe.

#### 3.1.3 PHE/Cambridge model

The PHE/Cambridge model (Birrell et al., 2021) is a multi‐type deterministic compartmental model (Section 2.2) with an age‐stratified structure in each of a number of spatially disjoint regions of England. The latent and infectious states are subdivided into two states each to give non‐exponential waiting times. The regional epidemics are assumed to be non‐interacting, but they have co‐dependence through some shared parameters. The model uses data on: COVID‐19 deaths; serological data from the NHS Blood Transfusion Service during the first pandemic wave, providing information on the presence of immunity conferring antibodies in blood sera samples; and contact matrices derived from the POLYMOD study (Mossong et al., 2008), updated weekly through Google mobility, the UK time‐use survey UKTUS (Gershuny, 2017) and information on school attendance from the Department for Education (DfE) (van Leeuwen et al., 2020). Deaths are linked to the new infections generated by the transmission model via the convolution of a time‐ and age‐varying fraction (the infection‐fatality ratio, IFR) of the infections over an assumed‐known distribution of the time from infection to symptoms and from symptoms to death. The proportions testing positive in the serological testing inform the fractions of individuals in the population remaining susceptible to infection, subject to the sensitivity and specificity of the test. The unknown parameters, estimated in a Bayesian framework, fall into two groups: those governing the transmission process and those related to the observational process. Some parameters, for example the time‐varying infection‐fatality rates and the serological test sensitivity and specificity, are ‘global’—that is, they are the same in each region—whereas the majority are regional parameters, such as the initial seeding of infection and the parameters that define the effective reproduction number $R_{e}(t)$.

**FIGURE 3:**
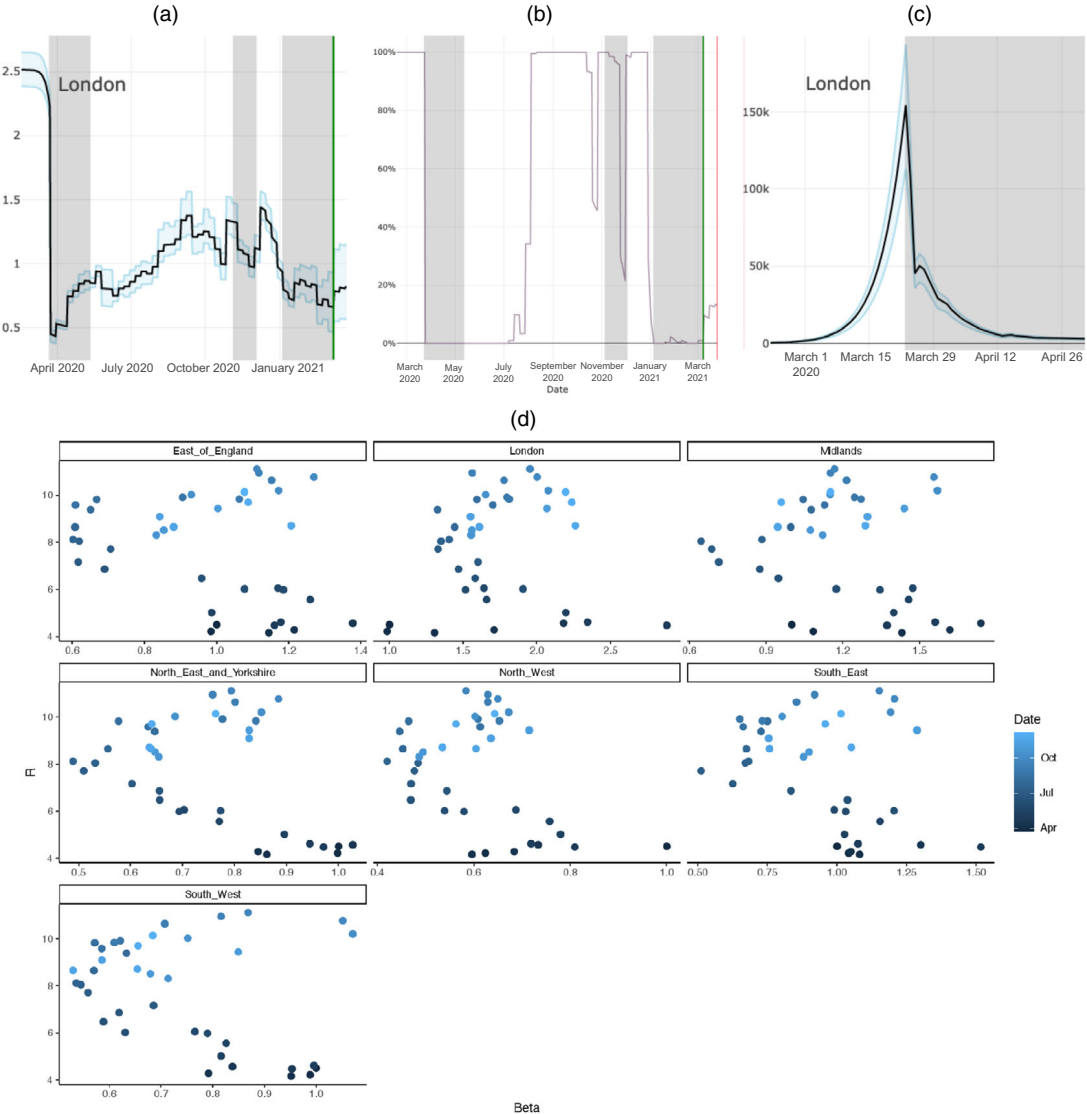
(a) PHE/Cambridge model: estimated $R_{e}(t)$ over time based on data up to 26 March 2021 for London; (b) the corresponding probability that $R_{e}(t)>1$ over time; (c) estimated incidence around the time of the first lockdown in March 2020; (d) The relationship between β(t) (x‐axis) and the dominant eigenvalue of contact matrices derived from POLYMOD, UKTUS, Google mobility and Department of Education data (y‐axis). The colour of points indicates the point during the pandemic, with darker points corresponding to the earlier stages. [Colour figure can be viewed at wileyonlinelibrary.com]

Specifically, the reproduction number is a product of a number of quantities: weekly contact matrices; contact parameters that account for mis‐specification of these matrices and have the interpretation of age‐group susceptibilities to infection given an infectious contact; time‐varying transmission potentials β(t); and region‐specific $r_{0}$ parameters. Initially, $R_{0}$ is calculated directly from these growth rates (Wearing et al., 2005). Over time, β(t) evolves through a random‐walk, with weekly increments, encapsulating changes in the nature of social interactions (such as social‐distancing, improved hand hygiene and mask‐wearing). The weekly changes in the contact matrices and β(t) induce consequent step changes in $R_{e}(t)$. With each step change, the system is shifted away from its equilibrium (if the equilibrium had even been found, see Section 2.2). So, how reliable is the value of $R_{e}(t)$ as derived from the dominant eigenvalue of the next‐generation matrix? The sharpest change in $R_{e}(t)$ followed the first lockdown in March, 2020. Figure 3a shows that this led to an estimated fall in $R_{e}(t)$ from 2.5 to less than 0.5. Figure 3b shows the certainty that at this time $R_{e}(t)>1$. However, after an initial steep drop, there is actually an increase in infections (Figure 3c), and there appears to be a lag of 3 days (the assumed mean length of the latency period) before incidence can be seen to be obeying an $R_{e}(t)<0.5$ regime. In practice, the weekly changes in $R_{e}(t)$ constitute much smaller perturbations, and $R_{e}(t)>1$ is a reliable indicator of increasing transmission. However, the potential exists around large‐scale pandemic interventions for the estimated $R_{e}(t)$ to be more volatile than the smoothed growth rates that can be derived directly from the time trend in the estimated infection incidence.

Incidence of COVID‐19 deaths is the primary data stream used in this model. The assumed time from infection to death has a mean of around three weeks, which, when coupled with the reporting delay inherent in deaths registration, results in these data being uninformative about patterns of incidence over the previous two weeks. Therefore, any estimate of $R_{e}(t)$ would appear to be a very lagged indicator. However, the weekly contact matrices are available almost in real‐time, and it is these data that are used to produce ‘nowcasts’ of $R_{e}(t)$—the projection forward of the lagged $R_{e}(t)$ estimates to the current day. As we have no information on the β(t) over this period, it is assumed that changes in mobility and school attendance, for example, translate directly into changes in $R_{e}(t)$ with no compensatory or amplifying effects. This has led to estimates for the reproduction number produced by the PHE/Cambridge model seemingly being out of step with many of the companion models contributing to the SPI‐M consensus at times of changes in pandemic mitigation measures or closure/re‐opening of schools. Figure 3d examines the validity of the assumption that $R_{e}(t)$ changes in proportion to the spectral radius of the weekly contact matrices, by plotting them alongside the estimated β(t) used to scale them. A negative correlation would indicate that the model seeks to counteract or downplay the impact of changes in mobility on transmission, whereas a positive correlation would suggest an amplification. From the plots it is clear that both correlations are present: there is a negative correlation over the pandemic first wave, but a positive correlation over the second half of 2020, suggesting more recent changes in transmission were more extreme than the mobility data might have suggested. The consequence of this can be seen in Figure 3a, where the negative correlation leads to a greater smoothing in the $R_{e}(t)$ as the impacts of any changes in mobility are dampened by the compensatory β(t). This makes sense as pandemic policy and governmental advice were consistent over this period. In the second wave, measures were imposed and relaxed not only with greater frequency, but asynchronously across the country, whilst schools were open with periods of holiday. Viewed in this way, it is perhaps less surprising that the $R_{e}(t)$ is more volatile over this period. Greater reliability in the now‐casting of $R_{e}(t)$ could therefore be achieved by expanding the model to incorporate such possible correlations, thus increasing the utility and reliability of movement and contact data to inform transmission.

### 3.2 Communication challenges

Since May 2020, the UK Government has published official estimates of $R_{e}(t)$ and, from the summer, $r_{e}(t)$, which conveys the rate of spread in real‐time rather than in generations of infectives (see Section 2). The alternative related measure of the doubling time does not have the same long history of R and r, but is also conceptually simple and easy to communicate during periods of epidemic growth. However, as a growing epidemic gradually slows down and starts declining, the doubling time tends to infinity and turns into a halving time, thus lacking an easy‐to‐understand threshold. Although the growth rate does have a threshold ($r_{e}(t)=0$), being a rate, it is harder to communicate.

The published estimates of $R_{e}(t)$ are produced on the basis of the contributed estimates from the SPI‐M modelling groups. The complexities of the full collection of estimates and their inherent uncertainties motivated an approach for the combination of model estimates (Maishman et al., 2021). Because of the differences in model structure and data streams used by the various modelling groups, as well as the limited interaction between them, model outputs were treated as independent expert ‘opinions’. The conceptual challenges associated with model combination have been discussed extensively in SPI‐M and include: the weighting methodology, giving equal weight to all contributing models, rather than using measures of model performance, a difficult process when different models generate different outputs and there is no observable ‘true’ value of $R_{e}(t)$; and the combination of estimates that are affected by different lags. More recently, SPI‐M have been putting forward lagged estimates for $R_{e}(t)$ as not all groups are able to nowcast the effective reproduction number to the present day. Whether or not the caveats have been fully conveyed, how these estimates are used to inform policy is also not straightforward. In one example, in results published in early June (Birrell et al., 2020‐06‐03), the PHE/Cambridge model produced an estimate of $R_{e}(t)$ centred on 1.01 for North West England, corresponding to a posterior probability of 51% that $R_{e}(t)>1$. This was the first publicly available regional estimate for $R_{e}(t)$ from a SPI‐M model, and was used as the justification for schools to remain closed in Greater Manchester (The Guardian, 6 June, 2020), contrary to national re‐opening policy. This local decision was clearly guided by a single, highly uncertain metric. Estimates of the prevalence of infection, and in particular hospital occupancy, as well as growth rates should be factored into such a decision. How to combine these indicators remains an open, and to some extent political, problem.

## 4 PRACTICAL CONSIDERATIONS AND OUR POSITION

The deceptively intuitive meaning associated with the different types of reproduction numbers masks a plethora of subtleties in their definition, interpretation, estimation and communication. In the simplest epidemic models, $R_{0}$ is often taken as the most useful quantity that, alone, informs about how parameter values affect model behaviour, as it determines the final size of the epidemic, the critical immunity threshold and the amount of transmission to be prevented in order to stop the outbreak. However, these properties are rapidly lost as model complexity increases. Furthermore, when studying an epidemic model theoretically, $R_{0}$ and its time‐varying counterparts are typically thought of as implying whether the number of infections will grow or decline. However, when assessing an epidemic in real time, parameters are estimated from surveillance data as they become available, so that reproduction numbers are estimated to be greater or smaller than one because the observed data are growing or declining. In addition, their value is used to measure how ‘fast’ the epidemic is growing or declining. However, the latter concept is to be interpreted in a generational perspective, while we argue that the same information is provided more directly by the exponential growth rates, which describe the epidemic speed in real time.

In this position paper we have presented and discussed the less commonly used control reproduction number $R_{c}(t)$. It is worth stressing that this is a unique value describing the impact of transmission of all current intervention measures combined. Although, when evaluating control measures, policy makers and the public may be interested in how each intervention affects the reproduction number, thus attempting to assign an ‘R budget’ to each measure, such an approach might be helpful in specific circumstances but is conceptually flawed, since the impacts of different interventions are not compounded in any simple fashion. The control reproduction number $R_{c}(t)$ only describes the overall potential impact on transmission of the combined interventions at time t, but has the purpose of comparing sets of control measures applied at different times under potentially different amounts of population immunity. Furthermore, we argue that, as COVID‐19 transitions from epidemic to endemic, $R_{c}(t)$ is also likely to facilitate the discussion around control when waning immunity starts to dominate the system dynamics.

Used as summary statistics, reproduction numbers and growth rates have strengths and limitations (Parag et al., 2022). We have highlighted challenges that apply to both:

- They are averages over time, where an arbitrary choice of the time window, which should be long enough to grant enough data points for estimation but short enough to capture the ‘latest’ trend, affects both the uncertainty around the point estimates and the time such estimates allegedly refer to.
- They conceptually pertain to an exponential phase, so although estimates can be computed during the transitions between different phases, their interpretation is unclear; this is particularly the case for multi‐type models, where continuously changing policies affecting contact patterns (e.g. lockdown, opening and closing schools, etc.) may cause the system to hop from transient phase to transient phase without ever stabilising in a regime where the proportions of cases of each type have converged to the component of the dominant eigenvector.
- When estimated from data, these quantities are necessarily delayed: infections are not observed directly, but rather reconstructed from other observable events in individuals' infectious episodes; latest data points are not always reliable; data collation is not instantaneous.
- All data streams arise from measurement and ascertainment processes which can change over time. Until enough data become available to capture such a change, inferred trends in R and r may be biassed. Challenges lie in balancing the input of earlier, less direct, data, with more robust but delayed data and reaching a timely understanding of how the observational process might be varying.

In addition to these difficulties, while r values can be directly inferred from data, reproduction numbers are predominantly estimated through a model that requires assumptions on the transmission process (e.g. at least the specification of a serial interval) and this additional transmission step makes them slower at responding to changes in observed data trends. For these reasons, we argue that the growth rate r is often preferable for asserting how quickly an epidemic is growing or declining, or whether a previously applied intervention is having a visible effect. On the other hand, R does provide information on how much transmission needs to be prevented to achieve controls, although the relevance of such information is unclear when the impacts of non‐pharmaceutical interventions are uncertain.

Ultimately, no single number can summarise all the relevant information about the epidemic and at best provides a limited, partial, perspective. Instead, a combination is needed: at the very least, metrics of both the current epidemic state and its trend. As an example, R=1 (or, equivalently, r=0) indicates that daily infections are sustained. However, this provides no information of whether they are stable at, for instance, 10 or 10,000 new infections per day, with very different outcomes in terms of public health response. In practice, policy response to an epidemic relies on a diverse toolkit of measures that, integrated together, are used to make risk assessment and take policy decisions. A key challenge remains how to communicate combinations of different metrics, rather than a single number, to both policy makers and the public, especially when different epidemic indicators may disagree with each other.

The COVID‐19 pandemic has brought infectious disease modelling and the reproduction number into public discourse. While the concept of R is important, it has grown to dominate the media, overshadowing r as a key metric of epidemic spread. One potential reason is the easy interpretation of the meaning and threshold value of R (a number) compared to the possibly less intuitive r (a rate). Doubling times, which are mathematically equivalent to exponential growth rates, can be easier to communicate when the epidemic is growing, but less so when switching between epidemic growth and decline. A second reason is related to the dominance of reproduction numbers over growth rates in the scientific literature. At the start of an epidemic, for instance, modelling studies typically strive to produce and present $R_{0}$ estimates as their main result, possibly driven by the fact that only reporting exponential growth estimates, though informative, does not appear as a worthwhile scientific result. However, the single numerical results often mask the fact that R estimates are inherently dependent on the underlying modelling assumptions used to analyse the data (Pellis et al., 2021), and, beyond their apparent simplicity, hide a range of subtle challenges with definition and meaning. We believe it is the responsibility of the scientific community to educate politicians and the general public as to the meaning and limitations of estimates of epidemic spread, and although at the beginning of this pandemic we were pleased to have introduced the concept of reproduction numbers to policy makers and the public, we encourage increased transparency in the communication of their estimates in combination with their underlying assumptions, precise meaning and limitations.

## FUNDING INFORMATION

All authors are supported by the UKRI through the JUNIPER modelling consortium (grant number MR/V038613/1). Lorenzo Pellis, Christopher E. Overton and Helena B. Stage gratefully acknowledge the Wellcome Trust and Royal Society (grant number 202562/Z/16/Z). Paul J. Birrell, Joshua Blake and Daniela De Angelis acknowledge the UKRI Medical Research Council, core Unit funding (MC UU 00002/11). Francesca Scarabel is also affiliated to the CDLab (Department of Mathematics and Computer Science, University of Udine), the Gruppo Nazionale per il Calcolo Scientifico (GNCS) of the Istituto Nazionale di Alta Matematica (INdAM), and the Research group “Modellistica socio‐epidemiologica” of the Unione Matematica Italiana (UMI). Helena B. Stage also acknowledges the Alexander von Humboldt Foundation for their support. Ellen Brooks‐Pollock and Leon Danon are supported by Medical Research Council (MRC) (MC/PC/19067). Leon Danon is supported by EPSRC (EP/V051555/1), The Alan Turing Institute under the EPSRC grant (EP/N510129/1) and investigator‐led grants from Pfizer on unrelated topics. Thomas A. House is supported by the Royal Society (grant number INF∖R2∖180067). Ian Hall is supported by the National Institute for Health Research Health Protection Research Unit (NIHR HPRU) in Emergency Preparedness and Response and the National Institute for Health Research Policy Research Programme in Operational Research (OPERA). Lorenzo Pellis, Thomas A. House and Ian Hall are also supported by The Alan Turing Institute for Data Science and Artificial Intelligence (under the EPSRC grant EP/N510129/1) and EPSRC (EP/V027468/1). Matt J. Keeling is supported by Health Data Research UK, which is funded by the UK Medical Research Council, EPSRC, Economic and Social Research Council, Department of Health and Social Care (England), Chief Scientist Office of the Scottish Government Health and Social Care Directorates, Health and Social Care Research and Development Division (Welsh Government), Public Health Agency (Northern Ireland), British Heart Foundation and the Wellcome Trust. Matt J. Keeling is affiliated to the National Institute for Health Research Health Protection Research Unit (NIHR HPRU) in Gastrointestinal Infections at University of Liverpool in partnership with UK Health Security Agency (UKHSA), in collaboration with University of Warwick. Matt J. Keeling is also affiliated to the National Institute for Health Research Health Protection Research Unit (NIHR HPRU) in Genomics and Enabling Data at University of Warwick in partnership with UK Health Security Agency (UKHSA). Jonathan M. Read is supported by the MRC (grant number MR/V028456/1). Daniela De Angelis and Ellen Brooks‐Pollock are also supported by the NIHR Health Protection Research Unit in Behavioural Science and Evaluation (grant number NIHR200877) and the NIHR Cambridge Biomedical Research Centre (BRC‐1215‐20014). The views expressed are those of the authors and not necessarily those of the NHS, the NIHR, the Department of Health and Social Care, Public Health England or the UK Health Security Agency.

## Data Availability

The data used in Figure 2 were obtained through a data sharing agreement with the National Health Service (NHS) England, and unfortunately cannot be provided. The data used in Figure 3 are not publicly available but it can be obtained upon request from the Public Health England Office for Data Release.
